# Longevity of continuous prosthetic finger control using implanted electromyographic signals without recalibration

**DOI:** 10.21203/rs.3.rs-11245807/v1

**Published:** 2026-10-18

**Authors:** Olivia G. Lee, Luis H. Cubillos, Hisham Temmar, Dylan M. Wallace, Aren I. Hite, Jesse C. DeMatteo, Joseph T. Costello, Jake J. Joseph, Madison M. Kelberman, Miranda G. Copenhaver, Theodore A. Kung, Deanna H. Gates, Paul S. Cederna, Cynthia A. Chestek

## Abstract

Background For decades, the field of robotic prosthetic hands has been hindered by a fundamental lack in effective, reliable control strategies. Currently, myoelectric hand prostheses are most often controlled with muscle activity recorded from the surface of the skin, or surface-electromyography (EMG). However, surface-based systems are mainly limited to grip control that requires frequent calibration. Methods In this study, we use EMG signals recorded from electrodes implanted in regenerative peripheral nerve interfaces and residual innervated muscles of the arm to decode continuous movements of two virtual finger groups in an able-bodied monkey and in a person with a transradial amputation. We compared the decoding performance of a linear and a nonlinear decoder against the participants’ natural hand performance during a two-finger random target matching task. To assess the reliability of this control, we measured the participants’ task performance over several months without recalibrating decoder parameters and investigated trends in online EMG-patterns when a static or same-day trained decoder was used. Results Here we demonstrate two critical milestones for prosthetic control simultaneously: high performance, continuous control of two finger groups and evaluate long-term stability of this control without decoder recalibration. Using a linear decoder, the monkey and the human performed the two-finger task with 63% and 56% task performance relative to their natural hand performance. Notably, when we tested these decoders without recalibration, we found that for both participants there was no significant decline in task performance (Monkey: p = 0.21, Human: p = 0.78) for several months (Monkey: 148 days, Human: 283 days). Interestingly, users adapted their muscle activation patterns to match the original decoder calibration, allowing for accurate finger control months after calibration even when the signals themselves changed day-to-day. Conclusions These results demonstrate that electromyographic signals recorded from intramuscular electrodes could serve as a stable neural interface for prosthetic control, and that long-term performance may depend on user adaptation rather than signal stability alone. Trial registration: Clinicaltrials.gov NCT03260400. Registered 25 July 2017.

**Additional Declarations:** Competing interest reported. The authors declare the following financial interests and personal relationships that may be considered as potential competing interests with the work reported in this paper. Dr. Cederna and Dr. Kung serve as President and Chief Medical Officer, respectively, of Blue Arbor Medical Technologies, a company that makes implanted devices for prosthetic control. Blue Arbor Technologies holds an exclusive option with the University of Michigan to license intellectual property invented by Dr. Cederna and Dr. Chestek (US Patent US10779963B2). This research was conducted in accordance with the principles embodied in the Declaration of Helsinki and in accordance with local statutory requirements. The human participant provided her written informed consent to participate in this study. She also consented to the use of her images in research publications.

## Background

More than 30 muscles provide the human hand with the ability to perform complex and dexterous movements every day [1]. For the millions of people worldwide with upper-limb loss, losing some or all these muscles can significantly affect their quality of life [2]. These individuals often struggle to perform simple activities of daily living [3], which can lead to increased healthcare costs [4], reduced ability to work [5], and a decline in mental health [6]. Advanced robotic prosthetics have been proposed as a potential solution for restoring dexterous hand function [7]. Research in this area has produced robotic hands capable of highly specific, continuous control of multiple degrees-of-freedom (DoFs), some with a rudimental ability to facilitate sensory feedback [8,9].

Despite these advancements, inadequate neural interfaces prevent end-users from gaining accurate and reliable control of these crucial additional DoFs [10]. Instead, advanced prostheses are used primarily to perform discrete grasps or single-DoF movements, such as opening and closing a hand [11–13]. This level of control can be useful for some simple, sequential tasks, but does not take full advantage of these devices' advanced functionality or approach full restoration of dexterous hand function. In large part due to such limited functionality, only 10–25% of people with upper-limb loss choose to use an advanced prosthesis [14–18].

The most common method used to control advanced prostheses today is with surface-electromyography (sEMG), electrical activity from muscles recorded with electrodes placed on the surface of the skin [11, 19] Although widely used in clinical practice and research applications, sEMG-based prosthetic control is not ideal. Surface electrodes can lose contact with the skin, shift positions during movement, or change in impedance due to perspiration, which can all result in the need for controller recalibration [20–22]. Frequent recalibration of sEMG-based controllers can contribute patient burden and subsequent prosthetic abandonment [23, 24]. In addition to frequent recalibration, sEMG signals recorded from the surface of the skin are often missing the detailed information needed to control complex finger or hand muscles, as those muscles are often buried deep within the limb or susceptible to crosstalk from neighboring muscles [25, 26].

An alternative to sEMG prosthetic control is to use EMG signals recorded from electrodes implanted into muscles via surgery. Implanted electrodes can access the contraction of deeper muscles and are less susceptible to nonstationarities that occur with surface electrodes [10, 25, 27]. In addition, reinnervation techniques such as targeted muscle reinnervation (TMR [26]) and regenerative peripheral nerve interface (RPNI [28]) surgery enable targeting muscles otherwise lost during amputation. Both strategies use the muscle tissue as a bio-amplifier for low-amplitude nerve signals, either through existing muscles (TMR) or muscle grafts (RPNI). With both approaches, electrodes can be implanted into reinnervated muscle tissue to provide relevant (and potentially otherwise unavailable) signals for prosthetic control [28–31]. Signals recorded from electrodes implanted in residual muscles and RPNIs have been shown to provide stable control of discrete grips over several months despite some session-to-session variation in SNR [32], but there has yet to be a long-term exploration of continuous, multi-DoF control such as simultaneous finger movements.

In this study, we demonstrate continuous control of two finger groups in an able-bodied monkey using electrodes implanted in the forearm, where all the muscles responsible for finger movements are present and true joint angles of the fingers can be measured. Being able to observe monkey's actual finger movements provides the ground truth behavior for comparison to the EMG signal recording. We also demonstrate continuous control of the same two finger groups for a person with a transradial amputation using implanted electrodes in her residual forearm muscles and previously created RPNIs. For this participant, all her intrinsic hand muscles are absent due to her transradial amputation and additionally, there is no access to ground truth behavior to compare to the EMG recording. Despite this challenge, both the monkey and the human were able to simultaneously control the two finger groups with their decoded intramuscular signals in real-time. We compared their online task performance to their baseline natural hand control and found that both the monkey and the human achieved substantial task performance relative to their natural hand control.

To assess the reliability of this control, we measured the participants' task performance over several months (148 days for the monkey, 283 days for the human), without recalibrating decoder parameters. Interestingly, we found that for both the monkey and the human participant, continuous finger control was maintained without any significant decline in task performance while using the static decoder parameters. Users achieved this stable control by seamlessly adapting their muscle activation patterns to the original decoder calibration, even when the signals themselves changed day to day. These results demonstrate that EMG signals from implanted electrodes could provide stable, continuous control of two finger groups without recalibrating decoder parameters. These findings address a historic bottleneck in this field of robotic prosthetic control: maintaining neural interfaces despite signal nonstationary over time. Our results further suggest that stability arises not solely from signal stability, but also from the user's ability to adapt their muscle activation to the pattern used to calibrate the decoder parameters. This work represents a significant step towards a more reliable neural interface for reliable, dexterous prosthetic hand control.

### Methods

## Subhead 1: Study design

This study follows a prospective, non-randomized design. Neither sex nor gender was considered in this study. The experiments were designed to evaluate the quality and reliability of continuous finger control with implanted-EMG. Based on previous literature, we hypothesized that implanted-EMG signals would be sufficient for decoding continuous movements of multiple DoFs, and that a high level of control could be maintained without recalibration. To test our hypotheses, we: (1) developed a similar two-finger random-target task for both a monkey and human participant, (2) trained decoders to predict finger movements using recordings from intramuscular electrodes, (3) measured decoder performance in real-time experiments, (4) compared decoder performance within session to the participants' natural hand control, and (5) analyzed decoder performance over several months without recalibration.

### Subhead 2: Monkey model

All surgical and experimental procedures with non-human primates were performed in accordance with NIH guidelines and approved by the University of Michigan's Institutional of Animal Care and Use Committee for Laboratory Animal Medicine. The monkey in this study (male, 12yo at time of implantation) is an able-bodied rhesus macaque. He has 8 bipolar electrodes implanted in his long flexors and extensors that control finger and wrist motion [33]. The bipolar electrodes are a modified version of the long-term electrodes used for the NeuRx (R) diaphragmatic pacing system (PMA Subject H00018, Synapse Biomedical, Oberlin, OH, USA).

### Subhead 3: Human participant

The human participant (female, 52yo at time of implantation) had a partial right-hand amputation because of necrotizing fasciitis. She experienced a significant amount of neuroma pain and phantom limb pain along with limited range of motion of her remaining fingers and wrist. She voluntarily underwent a distal transradial amputation with simultaneous RPNI surgery. One year later, she had electrodes implanted into two ulnar nerve RPNIs, one median nerve RPNI, and five residual innervated muscles as described previously [28]. The bipolar electrodes were a modified version of the long-term electrodes using the NeuRx (R) diaphragmatic pacing system (PMA Subject H00018, Synapse Biomedical, Oberlin, OH, USA). The electrodes were implanted under an Investigational Device Exemption for this study. The Institutional Review Board at the University of Michigan approved this study (HUM00124839), and the participant provided their written and informed consent. The authors have confirmed that any identifiable participant in this study have given their consent for publication.

### Subhead 4: Task design

The monkey was trained to sit in a chair (Crist Instruments, Hagerstown, MD, USA), with his head secured by customized titanium posts (Crist Instruments) and his percutaneous leads connected to a Cerebus neural signal processor (NSP, Blackrock Neurotech, Salt Lake City, UT, USA). His arms were secured in acrylic restraints with the elbows fixed at 90 degrees. The monkey's left hand was placed into a manipulandum with bend sensors that could measure relative position and velocity based on impedance changes. The manipulandum separates the monkey's fingers into the two groups with two hinged doors (index (IDX) finger in one, middle/ring/small (MRS) fingers in the other), each door fitted with a single bend sensor to measure the joint angle of that finger group. The manipulandum was calibrated so that output values ranged between 0 (full extension) and 1 (full flexion) of each finger group (Fig. 1a). The virtual fingers could be controlled in either hand-control mode via the manipulandum, or in closed-loop control via real-time EMG decoding (i.e., EMG signals converted to position updates for the virtual fingers). Hand-control mode is often described as “offline” or “open-loop” mode. Closed-loop control is commonly denoted as “online” or “decode” mode. The two-dimensional finger task is identical to the task developed by Nason et al [33]. The finger task required placing the virtual index (IDX) and middle/ring/small (MRS) finger groups on independent targets for a 750-ms during offline mode for training and 500-ms during online mode for testing (testing vs. training protocols are explained in a subsequent section). The targets were randomly chosen within the full range of motion for both offline training and online testing. The target size was 15% of the active range of motion. For offline calibration sessions, 400 trials were completed for decoder training, while 300 online trials were completed for each condition during closed-loop experiments broken up into in 150-trial “runs”. When the monkey completed trials, he was rewarded with a drink of apple juice. During the session, he was freed of distractions and head restrained.

The human participant sat in a chair with her residual limb resting on a table in front of her, while her percutaneous leads were connected to a Cerebus NSP. She faced a TV screen that displayed a virtual hand (Fig. 1b). The virtual fingers were controlled in auto-hand mode (i.e. directly controlled via computer-prompted trajectories) or in closed-loop. During auto-hand, or offline-mode, the participant followed the prompted finger positions on the screen with her phantom limb while EMG was recorded from her implanted electrodes. Due to limb loss, measurements of physical limb movement could not be made such as with the monkey, so virtual hand kinematics were relied upon for training. Computer generated prompts have historically been used for calibrating decoders with this participant rather than mirroring strategies [28, 29, 32]. During closed-loop control, the EMG signals were converted to position updates for the virtual hand via the decoders described below. The finger task for the human was similar to the monkey task and required placing the virtual IDX and MRS finger groups on the targets for 750-ms during offline mode and 500-ms during online mode. The target size was also 15% of the active range of motion, and the targets were randomly chosen within the full range of motion. Once the targets were acquired, new targets were presented. For offline calibration sessions, 30 trials were completed for decoder training, while 100 online trials were completed for each condition during closed-loop experiments. To measure the human’s natural hand performance baseline, separate experiments were performed during which she followed along with the auto-hand prompts with her intact contralateral hand while wearing a glove fitted with bend sensors along each joint (Cyber Glove Systems, San Jose, CA). The impedance of the sensors was used measure joint angles and calculate velocities of the fingers.

### Subhead 5: Signal processing

To record the EMG signals from each electrode, the percutaneous leads for both participants were connected to a Cerebus NSP via a customized connector from the manufacturer of the bipolar electrodes (Synapse Biomedical, Oberlin, OH, USA). The electrodes were connected to the data acquisition system through a splitter box receiving TouchProof connections, along with a ground reference electrode. For the monkey, this reference was attached by an alligator clip to his fixed head-post implanted into the skull as done in previous work with this monkey [33, 34]. EMG was sampled at 30 kHz (raw) with a digital offset removal high-pass filter (0.25 Hz applied by the pre-amplifier. These signals were down-sampled to 2 kHz and filtered between 100 and 500 Hz before being sent to a real-time computer (xPC Target, Mathworks, Natick, MA, USA) for real-time processing (1-ms real-time update rate via xPC Target). The signals were notch-filtered at all power harmonics within the band (120, 180, 240, 300, 360, 420, 480 Hz; 4th-order Butterworth). For the human participant, the ground electrode was connected via a gelled surface electrode placed on her C7 vertebrae as done in previous work with this participant [28, 29, 32]. The splitter box was then connected to a pre-amplifier via a ribbon cable and then relayed to the main data acquisition system via a fiber optic cable. EMG was sampled at 30 kHz (raw) with a digital offset removal high-pass filter (0.25 Hz) applied by the pre-amplifier. These signals were down- sampled to 2 kHz and filtered between 100 and 500 Hz before being sent to a real-time computer (xPC Target, Mathworks, Natick, MA, USA) for real-time processing (1-ms real-time update rate via xPC Target). The signals were also notch-filtered at 60 and 120 Hz (4th-order Butterworth).

For both the monkey and the human participant, the mean absolute value (MAV) was then extracted from the filtered signals in real-time to be fed into decoders and further analyzed offline. MAV was chosen as the feature for decoding to keep consistent with previous studies that used this signal pipeline for prosthetic control [32]. The xPC Target computer further averaged the single-ms MAV from each EMG channel into larger non-overlapping time bins (32-ms intervals for the monkey, 50-ms intervals for the human). Bin sizes of 32-ms or 50-ms are typical values used in the literature [34–36].

## Subhead 6: Kalman filter

The velocity Kalman filter (KF) is a linear decoder widely used in neuro-prosthetics that optimally combines a state transition model (using the current position and velocity as a state) with an observation model (that relates the kinematics to neural activity) to estimate the current position and velocity. These are modeled as linear functions assuming Gaussian noise. In this study, the KF tracks a state composed of the position and velocity of both finger groups (length of four) and uses the binned MAV of the EMG activity as observations of the state. The KF computes the optimal ratio between the finger dynamics versus the signal observations. The KF takes in the current bin of EMG MAV and the previous state and then outputs the predicted finger velocities. The KF was implemented with NumPy and trained as described in [37, 38], but here we did not use the ReFIT recalibration.

### Subhead 7: LSTM

The long-short-term memory (LSTM [39, 40]) network is a type of RNN that uses memory cells and gates to control what information to remember and what to forget. The LSTM has achieved state-of-the-art results in BCI finger movement predictions, outperforming other linear and nonlinear methods [38,40]. The specific LSTM architecture used throughout this paper was presented in Costello et al. [40], but using binned EMG MAV as the input feature rather than intracortical neural recordings. During training, a sequence length of 20 and zero-initialization for the hidden state were used for each sample. The final output of each sample sequence was used to compute the loss. During online decoding, the hidden state was stored in memory, such that the inputs at each timestep were the current neural bin and the previous hidden state. In this work, all models were trained using PyTorch 2.6.0, AdamW, and a learning rate scheduler. For each offline and online testing day presented, the first 70% of the data was used for training, next 10% used for validation, and final 20% used for testing. For each online day, data was split instead into 80% training and 20% validation. The model parameters that resulted in the lowest loss on the validation set during training were used for evaluation on the testing set. When testing the LSTM with the human, we used a real-time system based off the open-source real-time system called BRAND (Backend for Realtime Asynchronous Neural Decoding) [41] instead of xPC.

### Subhead 8: Testing protocols

To quantify closed-loop EMG control, we gave both participants visual feedback of decoder output during their respective behavioral tasks. For each closed-loop experimental session, the participants began by completing a set of trials (400 for the monkey, 30 for the human) in offline mode. We then trained a decoder using the synchronized finger kinematics and EMG. Subsequently, we used the decoders to predict finger movements in real time where the virtual fingers had to remain within the targets for 500-ms for a successful trial.

For the monkey, offline decoder performance was measured by testing decoders on hand-control trials from 37 of the 42 days that $KF_0$ was tested online. The last 5 days of this set were excluded from this offline analysis because the FEA was swapped for a refurbished part during those sessions and introduced further signal biases. The same-day trained online decoder comparison was performed on a set of sessions where the same model architecture between the monkey and human participants was used (Hand: N = 5 sessions, KF: N = 3 sessions, LSTM: N = 3 sessions) While these decoders were tested online during more sessions than this set, the comparison was performed only on days where the LSTM architecture matched what was used for the human experiments. Throughout this paper, the “cross-day” or “static” decoder will refer to the decoder initially calibrated with parameters that were locked for all future online testing days. Static decoders are denoted with a subscript zero. On every day that the static decoders were tested, a new, “same day” decoder was also trained and tested, denoted throughout this paper by a subscript *k*, representing the Day k it was trained and tested on. Online testing of the cross-day KF ($KF_0$) for the monkey was performed during 42 sessions spanning 148 days total, where $KF_0$ was compared to $KF_k$ within each session. Online testing of the cross-day $LSTM_0$ was performed during 5 sessions spanning 23 days total, where $LSTM_0$ was compared to $LSTM_k$ within each session.

For the human participant, decoders were tested offline using hand-control trials from 8 days, which includes all the days that the decoders were tested online. Online comparison of same-day trained decoders was performed on all the sessions that the participant performed the finger task with her contrateral hand (Hand: N = 3 sessions), a same-day trained KF (KF: N = 7 sessions) and with a same-day trained LSTM (LSTM: N = 3 sessions). The LSTM was only tested on three sessions because it was difficult and frustrating for the participant to use. For this reason, it was also excluded from the cross-day decoder testing. Online testing of the cross-day $KF_0$ was performed during 7 sessions spanning 283 days total, where $KF_0$ was compared to $KF_k$ within each session.

To reduce confounders when comparing decoders, decoders were tested online using A-B-A-B blocking, where each block (A or B) is a set of trials using either decoder being compared (150 for the monkey, 50 for the human) for each run. For the monkey, the first 10 trials with each decoder were discarded in the online analysis. For the human participant, the first 5 trials with each decoder were discarded as there were fewer trials total.

## Subhead 9: Decoder analysis

Offline decoder performance was characterized by the Pearson correlation (*r*) coefficient, or “correlation”, and the mean-square-error (MSE) between the ground truth ( $y$) and the predictions ( $\hat{y}$) as described in the equations below.

$$r = \frac{\sum_t \left(y_t - \bar{y}_t\right)\left(y'_t - \bar{y}'_t\right)}{\sqrt{\sum_t \left(y_t - \bar{y}_t\right)^2 \sum_t \left(y'_t - \bar{y}'_t\right)^2}}$$

$MSE = \frac{1}{N}\sum_t \left(y_t - y'_t\right)^2$ These values were averaged across the two finger groups to obtain an *r* and MSE value for both position and velocity. Online decoder performance was characterized by Fitt’s law throughput given below, which accounts for both task difficulty and the time needed for completion (measured in bits/s). The variable $D_i$ is the distance of the $i^{th}$ virtual finger to the center of the $i^{th}$ target at the start of the trial, $R$ is the target radius (equal in both fingers), and $t_{acq}$ is the time to reach the target *(42)*. While throughput was the primary online performance metric, trial success rates and acquisition times are also reported. Success rate is computed as the ratio of the successful trials to the total number of trials, and acquisition times was time taken acquire both finger targets minus the hold time. Only successful trials were used to calculate mean throughput and acquisition times, while failed trials were accounted for in the overall success rate.

$$\text{Throughput} = \frac{\sum_{z=1}^{k} \log_2\left(\frac{D_i}{R} + 1\right)}{t_{acq}}$$

## Subhead 10: EMG analysis

To better understand the participants' control strategy, the root-mean-squared (RMS) of the EMG was calculated as done previously [28]. For this analysis, the EMG RMS was calculated on each channel and averaged over all trials of interest. To compare how the EMG was changing day-to-day, the EMG RMS was calculated for offline calibration trials from days that the cross-day KF decoders were tested online (Days 38–42 were excluded for the monkey for this analysis because the NSP front-end-amplifier was modified). To compare how participants were adapting their control strategy, the EMG RMS was also calculated for closed-loop trials during sessions where either a cross-day or a same-day trained decoder was used. To quantify differences in activation for open-loop and closed-loop trials, the deviation from a designated offline baseline (calibration trials on Day 0 or Day k) was computed as the absolute difference between the online RMS and offline RMS, divided by the offline RMS. To be specific, a value for EMG RMS was calculated for each channel for each control mode (offline or online) and for each decoder type (cross-day or same-day trained). The Manhattan (L1) distance was computed between the channel EMG RMS values from offline calibration trials and online trials where either a static or same-day trained decoder was used. The distances were then normalized between 0 and 1 by the average EMG RMS value for the offline calibration trials. These normalized distances between activation patterns were then compared for both participants.

## Subhead 11: Statistical analysis

Offline comparison of decoding algorithms used a Wilcoxon rank sum test. Offline performance metrics (correlation, MSE) are reported as mean value ± standard error of the mean (SEM). For online decoder performance, session-level comparisons were performed with a Wilcoxon signed-rank test between the matched sessions, with one mean per session. Performance metrics are reported as mean value ± standard deviation. Trends in decoder performance and EMG RMS were assessed with a linear regression model. A non-zero slope, i.e. change in decoder performance or mean offline EMG RMS over time, was evaluated with an F-test. If a significant change was detected, the sign of the slope was then determined an increasing or decreasing trend over time. Wilcoxon signed-rank testes were applied to assess if the median differences in activation patterns differed from zero, while two-sided Mann-Whitey U tests were used to directly compare the normalized distances between activation patterns under both baseline conditions. All statistical comparisons were analyzed with a significance level of $\alpha = 0.05$.

### Results

## Subhead 1: Continuous, multi-finger movements predicted with intramuscular EMG

Previous work has demonstrated continuous control of single finger movements with linear-regression-based decoders in able bodied monkeys using EMG signals recorded from electrodes implanted in finger muscles and previously created RPNIs [35]. We expanded on this by reconstructing continuous movements of two individuated groups of fingers with EMG recorded from implanted electrodes in both a monkey and a human participant. Taking cues from the related field of neural decoding for braincomputer interfaces (BCIs), we tested two decoders representing the state-of-the-art linear and nonlinear approaches: the velocity Kalman Filter (KF) [34, 35] and the long short-term memory recurrent neural network (LSTM) [39, 40]. Both the monkey and the human performed a task where they acquired virtual targets with virtual fingers shown on a screen in front of them (Fig. 1). In "hand-control" (offline) trials, the monkey moved his fingers within a manipulandum that measured the angles of his index (IDX) and middle-ring-small (MRS) finger groups. The manipulandum separates the monkey's fingers into the two groups with two hinged doors, each door fitted with a single bend sensor to measure the joint angle of that finger group. These measurements directly controlled the virtual fingers. The human participant followed along with computer-prompted trajectories of the virtual hand using her phantom limb to acquire the targets for the IDX and MRS finger groups. The kinematics of the monkey's actual fingers and the prompted finger trajectories for the human were used as the ground truth behavior for each and were recorded synchronously with the participants' EMG. For each calibration session where the participants performed a set of offline trials, we trained instances of the KF and the LSTM using the recorded EMG and ground truth behavior (37 sessions with the monkey (400 trials each session), 8 sessions with the human (30 trials each session)). We measured average prediction accuracies between the prediction and ground truth on all sessions with the Pearson correlation coefficient (aka "correlation") and mean squared error (MSE). On average, decoder predictions with either the KF or LSTM had correlations greater than 0.79 for both participants. These strong correlations between the ground truth and decoded position suggest that the intramuscular EMG signals contain sufficient information to predict finger movements. The mean offline decoder metrics for all sessions are shown in Table 1 for the monkey and human.

Table 1
**Offline decoder performance.** Complete offline decoded position results where each column header denotes the participant type (monkey or human) and the decoder type (KF or LSTM). A decoder was trained and tested on hold out sets from each day that a calibration session was performed (37 sessions with the monkey (400 trials each session), 8 sessions with the human (30 trials each session)). The metrics are averaged for the position predictions of both DoFs (mean ± SEM) for all decoders.

| Metric | Monkey | | Human | |
|---|---|---|---|---|
| | KF | LSTM | KF | LSTM |
| Correlation | 0.868 ± 0.037 | 0.927 ± 0.021 | 0.871 ± 0.097 | 0.799 ± 0.108 |
| MSE | 0.022 ± 0.009 | 0.010 ± 0.003 | 0.039 ± 0.038 | 0.075± 0.041 |

### Subhead 2: Multi-finger control recovered via online EMG decoding

While the offline analysis demonstrated that EMG signals from implanted electrodes contain sufficient information to predict finger movements, real-time prosthetic control requires that these predictions translate into effective closed-loop behavior. To simulate prosthetic control, participants also performed the random-target two-finger task in a closed-loop, “online” context, where the virtual hand was only controlled by real-time decoder predictions given the muscle activity. Online task performance was compared to each participant’s respective “natural hand performance”, or their performance with their actual hand (monkey) or intact contralateral hand (human). We defined this comparison of closed-loop control to hand-control as the relative performance (%) of their natural hand performance. The participants performed the random-target two-finger task in both hand-control and in a closed-loop control for several sessions. Task performance was measured by trial completion time (in seconds), success rate (in % successful trials), and throughput (in bits per second, bits/s), a metric that balances task difficulty and acquisition time (calculated using Fitt’s Law; see Methods). Trial metrics for both participants for multiple sessions are shown in Fig. 2 and summarized in Table 2. When using his hand to perform the task, the monkey achieved a mean success rate of 99% and an average throughput of 4.1 ± 2.2 bits/s. In closed-loop control, the monkey achieved an average of 63% and 76% of in terms of throughput relative to hand-control while completing the task with a KF and an LSTM, respectively. Example traces of the monkey using his hand and a KF to acquire targets are shown in Fig. 2b.

Table 2

**Online decoder performance.** Complete online task performance where each column header denotes the participant type (monkey or human) and the control type (Hand, KF, LSTM). The task metrics shown are success rate (in % successful trials), trial completion time (in seconds), and throughput (in bits per second, bits/s). The metrics are averaged across all trials for all sessions (mean ± standard deviation).

| **Metric** | **Monkey** | | | **Human** | | |
|---|---|---|---|---|---|---|
| | **Hand** | **KF** | **LSTM** | **Hand** | **KF** | **LSTM** |
| Success Rate (%) | 100 ± 0.2 | 98 ± 1.5 | 99 ± 2.0 | 97 ± 1.4 | 90 ± 6.2 | 69 ± 16.3 |
| Trial Time (s) | 0.29 ± 0.19 | 0.86 ± 0.45 | 0.80 ± 0.37 | 1.4 ± 0.77 | 2.5 ± 1.6 | 6.2 ± 0.52 |
| Throughput (bits/s) | 4.1 ± 2.2 | 2.6 ± 1.5 | 3.1 ± 1.9 | 1.5 ± 0.87 | 0.84 ± 0.51 | 0.59 ± 0.075 |

After demonstrating real-time finger control in the monkey where we had access to true behavior, we asked how this would translate to a more realistic use-case for the human participant who does not have a hand, and if similar proportions of hand-control could be achieved in closed-loop control. To get a baseline for her hand-control performance, the human participant performed the task with her intact contralateral hand while wearing a motion tracking glove. With her intact hand, she achieved a mean trial success rate of 99% and a mean throughput of 1.5 ± 0.87 bits/s. With the KF in closed-loop control, she achieved a mean trial success rate of 90%. She recovered an average of 56% and 39% of her native hand performance in terms of throughput while completing the task with a KF or an LSTM, respectively. Example traces of the human using her hand or a KF to complete the task are shown in Fig. 2d. Notably, the closed-loop task performance relative to native-hand performance is similar for the monkey and human participants with a simple linear decoder (KF). However, while the nonlinear LSTM significantly improved real-time finger control for the monkey from the KF ($p < 0.05$), the LSTM performed significantly worse than the KF for the human ($p < 0.05$) with an average trial success rate of only 69%. This interesting difference between using an LSTM with the monkey and the human may be the result of a lack in ground truth labels. Poor kinematic labeling may be less of an issue with a linear decoder like the Kalman Filter, which may offer a simpler or more predictable control strategy to the participant. This observation is further explored in the Discussion. Overall, these results demonstrate that EMG signals from implanted electrodes can provide continuous, multi-DoF control approaching native-hand task performance, even for a person with an amputation.

## Subhead 3: Multi-finger control for months without recalibration

Because sEMG-based prosthetic-control requires frequent recalibration [24] and implanted-EMG control has only been tested without recalibration for discrete grasps [32], we next examined the stability of continuous, closed-loop control with EMG signals from implanted electrodes. To test this, we trained decoders for each participant during an initial calibration session ($KF_0$ and $LSTM_0$ for the monkey, $KF_0$ for the human participant), and had both participants use these static decoders without recalibration during several sessions over the following months (Monkey: $KF_0$ used in 43 sessions over 148 days, $LSTM_0$ used in 5 sessions over 23 days; Human: $KF_0$ used in 7 sessions over 283 days). As LSTM's could not reach high-performance for online control within a single session (see Results Section 2) for the human participant, they were not tested over time. Per-session performance was measured using average throughput and acquisition time for both participants' cross-day decoders ($KF_0$ and/or $LSTM_0$), and a linear regression was fit over time to evaluate performance trends. We found no clear decline (n.s. slope) in task performance for any decoder with either participant (Fig. 3a, c) (see Fig. S1 for $LSTM_0$ results with the monkey). During each session that the cross-day decoders were tested online, we also tested decoders trained on data obtained during the session (same-day decoders, $KF_k$ and/or $LSTM_k$). Online task performance was measured for both the cross-day and same-day decoders and compared to each other (Fig. 3b, d). Across all sessions, the monkey's cross-day decoders performed with 1.2% higher throughput and 6.0% lower acquisition times than the same-day decoders on average, though that difference was not significant ($p > 0.05$). For the human participant, the cross-day decoder performed with a 9.8% lower throughput and 8.6% higher acquisition time on average across all days ($p < 0.05$). This was a small although significant difference in performance between the cross-day and same-day trained decoders. While the static decoder remained usable, same-day recalibration provided a slight benefit. Still, these results demonstrate that EMG recorded from intramuscular electrodes can provide reliable continuous control of two DoFs for months without recalibration, as no clear decline in task performance was observed during the available sessions.

## Subhead 4: Temporal variations in offline decoder performance

While cross-day decoders remained stable over months of online testing for both participants, we observed major differences in the offline decoder performance over time. When tested offline on held-out sets from the online testing days, the cross-day decoders showed significant day-to-day variations in prediction error for both participants (Fig. 4a, b). To examine the discrepancy between offline and online decoder performance, we measured the root mean square (RMS) of the EMG produced by the participants during all hand-control sessions on all channels. Figure 4 (c, d) shows this for an example flexor channel (FCR), demonstrating significant day-to-day variability in mean EMG RMS for both participants. A linear regression fit to the EMG RMS values across days for the two example channels showed no significant slope for either participant (Monkey: slope = 0.7uV/day p = 0.55; Human: slope = 0.3uV/day, p = 0.34). We observed similar results on all other channels for both participants, with significant day-to-day variability on each, but no significant slopes in EMG RMS over time (Table 3). These results agree with previous findings [32], that while there was no significant degradation in signal strength overall, day-to-day variability in EMG amplitudes was present. This is a likely contributor to the variation in offline performance of the static decoder across days.

Table 3
**EMG RMS trends.** Summary of EMG RMS linear regression statistical analysis across days where the columns denote the participant type (monkey or human). The next column headers denote the electrode channel location, the slope of the linear regression trend, and the p-value of the slope when tested for significance. For the monkey, values for the FCU channel are not included due to electrode breakage.

| **Monkey** | | | **Human** | | |
|---|---|---|---|---|---|
| Channel | Slope (μV/day) | p-value | Channel | Slope (μV/day) | p-value |
| FCR | 0.70 | 0.551 | EPL | 0.33 | 0.541 |
| FDP-1 | 0.00 | 0.976 | EDC | 0.53 | 0.559 |
| FDP-2 | -0.11 | 0.417 | FPL | 0.16 | 0.661 |
| FDP | -0.29 | 0.420 | FDP | 0.64 | 0.374 |
| FCU | - | - | Median RPNI | 0.07 | 0.543 |
| ECRB | 0.46 | 0.225 | Ulnar RPNI-1 | 0.07 | 0.285 |
| EIP | 0.07 | 0.812 | Ulnar RPNI-2 | 0.24 | 0.222 |
| EDC | -0.22 | 0.652 | FCR | 0.30 | 0.335 |

## Subhead 5: Users adapt their activation to match decoder calibration

We sought to understand how it was possible that the decoder performance remained stable online, even though offline performance varied day-to-day. The determine the source of this discrepancy, we first observed the EMG produced by the monkey during similar offline and online trials. Generally, we noticed that his activation during closed-loop control looked like his EMG from the offline trials that were used to calibrate the active decoder. An example of this observation is demonstrated in Fig. 5a, where the monkey's EMG in offline and online contexts are shown. In examples like this, we noted that when the monkey used the cross-day decoder for online trials several days after it was calibrated, the EMG looked similar to the activation from offline calibration trials from Day 0. Inversely, we noted that when the monkey used a same-day trained decoder on a later date (Day k), his activation looked more like calibration trials from that day than from Day 0. We observed examples like this for several trial motifs across all days, which led us to suspect that the monkey was adapting his control strategy to match the pattern of activation from the calibration trials of each decoder.

To quantify this observation for both participants, we calculated the distance of their EMG pattern of activation between offline and online contexts for all days. We first established a baseline pattern of activation for calibration trials on Day 0 and each Day k by taking the average EMG RMS of each channel. We also measured the average EMG RMS of each channel for all online trials where either $KF_0$ or $KF_k$ was used. Within each day, we took the difference of the channel EMG RMS values between the calibration baseline and the online patterns for both decoders. The deviations of the online EMG RMS values from the offline baseline values were then summed across channels and normalized between 0 and 1 by the average offline baseline for that day. These normalized distances between activation patterns were then compared for both participants, as shown in Fig. 5b, c.

This analysis demonstrated that when participants used the cross-day decoder online, their EMG-pattern was more similar on average to their baseline EMG pattern from Day 0 than to the baseline pattern on that later Day k. Similarly, when participants used same-day trained decoders, their online EMG-pattern was more similar to the baseline EMG-pattern from that Day k than from Day 0. This trend was significant for the monkey and less strong for the human participant, likely because there were fewer trials overall. This result suggests that users may be adapting their control strategy to compensate for signal variation and maintain stable online performance without recalibrating decoder parameters.

## Discussion

This study demonstrates that EMG recorded from implanted electrodes could provide more stable, continuous control of two individual finger groups for months without recalibrating decoder parameters. These findings address a central barrier in neuro-prosthetics: maintaining reliable neural interfaces despite signal nonstationarities over time. Our results further suggest that control stability arises not solely from signal stability, but also in part due to user adaptation.

We show that with EMG signals recorded from implanted electrodes, the monkey and the human performed the two-finger task with 63% and 56% task performance relative to their natural hand performance with a linear decoder (KF) (Fig. 2). The literature has shown that intramuscular EMG could be useful for controlling even more DoFs in the hand, such as thumb or wrist movements [25, 28]. More DoFs will continue to make greater use of the available prosthetic technology and potentially improve the overall user experience. Notably, we found that this closed-loop continuous finger control was maintained over several months without recalibrating decoder parameters (Fig. 3) despite significant variation in offline decoder performance (Table 3, Fig. 4). Previous studies have looked extensively into the nonstationarities that contribute to a loss in performance and reliability of surface-EMG control [20–22]. It could have been that intramuscular signals are simply more stable than surface-EMG, in addition to having higher signal-to-noise ratios [28]. However, this work agrees with previous findings that while day-to-day variation in implanted EMG-signals is observed, the signals remain useful for control without recalibration [29]. User-adaption may compensate enough for these variations to maintain online, closed-loop performance (Fig. 5).

For the monkey where we had access to ground truth behavior for decoder training, we show that the LSTM significantly improved upon the KF for finger control (Fig. 2). This result aligns with literature in the related field of neural decoder for BCIs which has shown that nonlinear approaches like recurrent neural networks (LSTM) can greatly improve overall decoding performance [38–43]. Interestingly, when the human participant used a nonlinear decoder her online trial success rate dropped from 90% with the KF to 69% with the LSTM (Table 2). Unlike with the monkey, we do not have access to true behavior for a person with limb loss, a significant challenge for training controllers that are well-aligned with the user's actual movements. This is the same challenge in the broader field of neural decoding for BCIs where true behavior is not available for decoder training. Recent research suggests that other loss functions and time-alignment methods can improve this issue [44, 45], but it remains to be seen if they are effective in real-time motor decoding for prosthetic control.

This work is a significant step towards reliable, high-performance multi-DoF control of advanced robotic prosthetics. However, clinical translation will require validation of the technology in and out of experimental settings. While throughout this study the decoders did not need to be recalibrated within session for users to perform the 2-DoF task at a high capacity, they were still bound by percutaneous leads during these experiments. For complete translation of emerging control strategies like this to be functionally validated, they need to be made accessible with commercially available, wireless systems that can be adapted for specific use-cases and deployed in clinical studies. These systems, should they be developed, could provide prosthetic users with stable, continuous multi-DoF control in their own homes for everyday activities.

## Declarations

#### Acknowledgments

We thank Eric Kennedy experimental support and animal expertise with the monkey. We thank Kayla Russell-Bertucci for experimental support with the human participant.

#### Funding

National Institutes of Health grant 1R01NS134834 (OGL, LHC, DMW, AIH, JCD, MGC, CAC, DHG, PSC, TAK)

National Institutes of Health grant 3U41NS129436 (OGL, MMK)

National Institutes of Health grant 5T32GM141746 (JJJ)

National Science Foundation grant EFRI/BRAID 2223822 (HT)

National Science Foundation grant DGE-2241144

**Author contributions**

Conceptualization: OGL, HT, CAC

Data acquisition: OGL, HT, LHC, DMW, AIH, JCD, MGC, JJJ, MMK

Data analysis techniques: OGL, LHC, AIH, JTC

Interpretation of results: OGL, LHC, DMW, HT, AIH, JTC, JCD, MGC, JJJ, MMK, CAC, DHG, PSC, TAK

Funding acquisition: CAC, PSC

Surgery design and procedure: PSC, TAK

Writing – original draft: OGL

Writing – review & editing: OGL, LHC, DMW, HT, AIH, JTC, JCD, MGC, JJJ, MMK, CAC, DHG, PSC, TAK

**Competing interests**

The authors declare the following financial interests and personal relationships that may be considered as potential competing interests with the work reported in this paper. Dr. Cederna and Dr. Kung serve as President and Chief Medical Officer, respectively, of Blue Arbor Medical Technologies, a company that makes implanted devices for prosthetic control. Blue Arbor Technologies holds an exclusive option with the University of Michigan to license intellectual property invented by Dr. Cederna and Dr. Chestek (US Patent US10779963B2). This research was conducted in accordance with the principles embodied in the Declaration of Helsinki and in accordance with local statutory requirements. The human participant provided her written informed consent to participate in this study. She also consented to the use of her images in research publications.

**Availability of data and materials**

The source data generated in this study will be provided with this paper as supplementary data with the manuscript and will be made available on the lab website [https://chestekresearch.engin.umich.edu/data- and-resources/]. The raw datasets used for this study are too large to be publicly shared, yet they are available for research purposes from the corresponding author on reasonable request.The Python code for the neural networks will be made available on the lab website as well.

## Figures

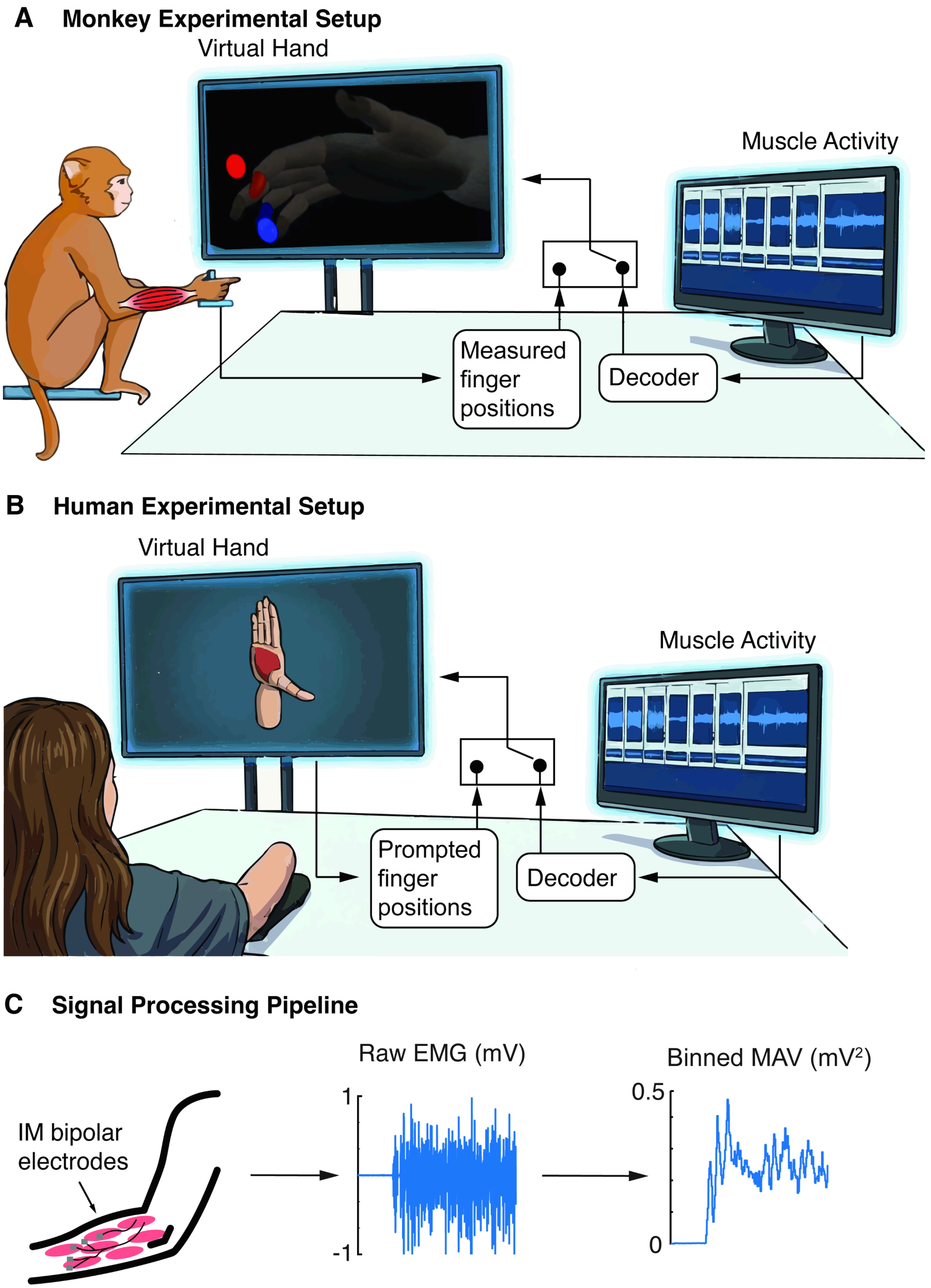

**Figure 1**

**Experimental setup.** (**A**) Monkey experimental setup. During open-loop trials, the monkey used a finger manipulandum (an apparatus with two independently movable, hinged doors fitted with bend-sensors) to control a virtual hand and acquire targets with visual feedback from the screen. Targets were randomly chosen along the arc of finger flexion for two finger groups (index (IDX) and middle/ring/small (MRS)). During closed-loop trials, the decoded finger positions controlled the virtual hand. (**B**) Human experimental setup. During open-loop trials, the participant followed finger positions prompted by a virtual hand on a screen using her phantom limb. Targets were randomly chosen between full extension and full flexion for the same finger groups as the monkey participant (IDX and MRS). (**C**) Muscle activity (EMG) was recorded from implanted bipolar electrodes (Monkey: 8 electrodes implanted in his long flexors and extensors that control finger and wrist motion; Human: 5 electrodes implanted in her long flexors and extensors that control finger and wrist motion, 2 electrodes implanted in ulnar nerve RPNIs and 1 electrode implanted in a median nerve RPNI. Mean-absolute value (MAV) was calculated in real-time and averaged over discrete time intervals, or bins (32-ms bins used with the monkey, 50-ms bins used with the human). The MAV was then fed into a decoder during closed-loop trials, which predicted finger kinematics in real-time.

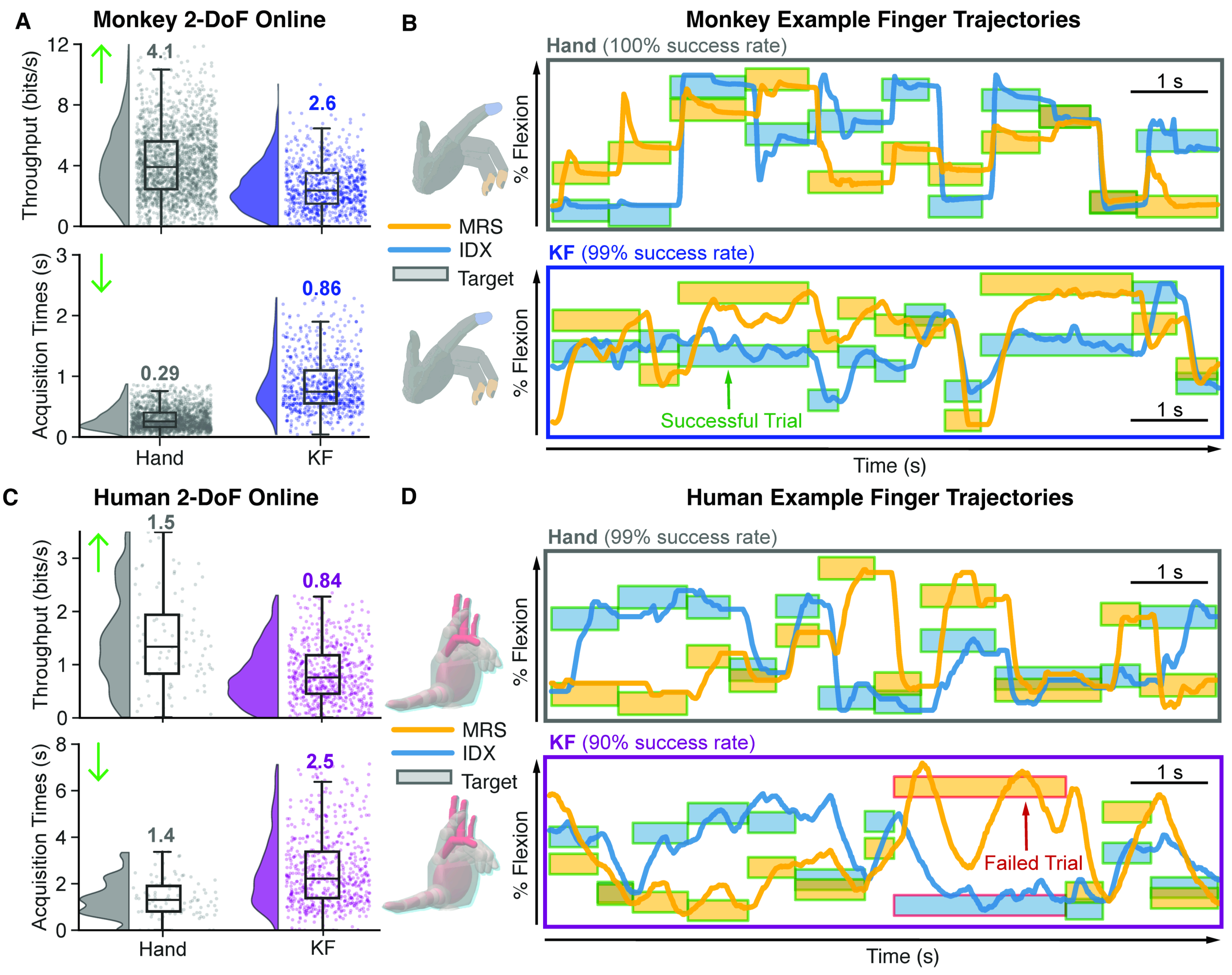

### Figure 2

**Continuous finger control recovered via real-time EMG decoding.** (**A**) Monkey online KF performance (N = 3 sessions, approximately 150 trials each session) compared to hand-control (N = 3 sessions, approximately 400 trials each session), measured by throughput (higher is better) and trial acquisition time (lower is better). The green arrows denote the direction of improvement. The boxes denote the IQR (whiskers denote 1.5 x IQR). All decoders were trained and tested on the same day for the 2-DoF random-target task. (**B**) Example monkey finger traces while using his hand and a KF decoder to complete trials. Boxes represent the target position for each finger, and the lines represent the finger positions. While slower to complete trials than his native hand, the monkey completes trials with the KF with 99% success rate. (**C**) Human online KF performance (N=7 sessions, approximately 100 trials each session) compared to hand-control trials completed with her contralateral intact hand from days (N=3 sessions, approximately 100 trials each session) while wearing a motion-tracking glove. All decoders were trained and tested on the same day for the 2-DoF random-target task. d, Example traces of the human participant completing trials with her intact-hand or during online-control with a KF, achieving success rates of 97% and 90%, respectively.

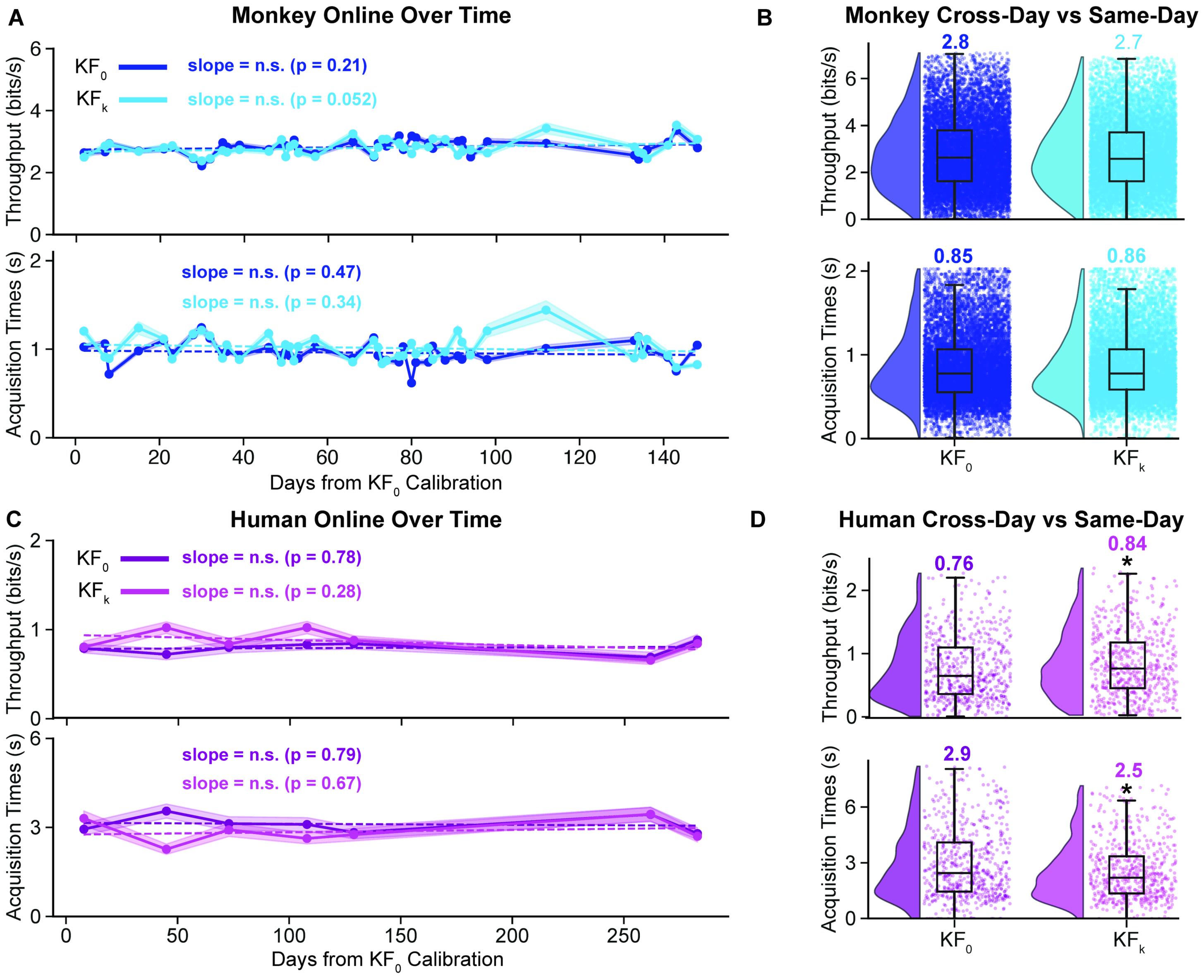

**Figure 3**

**Stable finger control without recalibration.** (**A**) Average values for throughput and trial acquisition time for each session are plotted for the static ($KF_0$) and same day trained ($KF_k$) decoders for the N=43 sessions that they were tested over 148 days. A linear regression was fit over all sessions denoted by the dashed line, and the regression slope was tested for significance (p-values labeled on plot). There was no significant slope in throughput or trial acquisition time when the monkey used $KF_0$ or $KF_k$ for N=43 sessions over 148 days. (**B**) Population data for the monkey's cross-day and same-day trained KFs. There were no significant differences in the monkey's task performance when he used $KF_0$ or $KF_k$ ($p > 0.05$). The boxes denote the IQR (whiskers denote 1.5 x IQR). (**C**) Average values for throughput and trial acquisition time for each session are plotted for the cross-day ($KF_0$) and same-day trained ($KF_k$) decoders for the N=7 sessions that they were tested over 283 days. A linear regression was fit over all sessions denoted by the dashed line, and the regression slope was tested for significance (p-values labeled on plot). There was no significant slope in throughput or trial acquisition time when the human participant used $KF_0$ or $KF_k$ for N=7 sessions over 283 days. (**D**) Population data for the human's cross-day and same-day trained KFs. Across all sessions, the human participant performance was slightly better on average with the same-day trained decoders than with the static decoder in terms of throughput and acquisition time ($p < 0.05$).

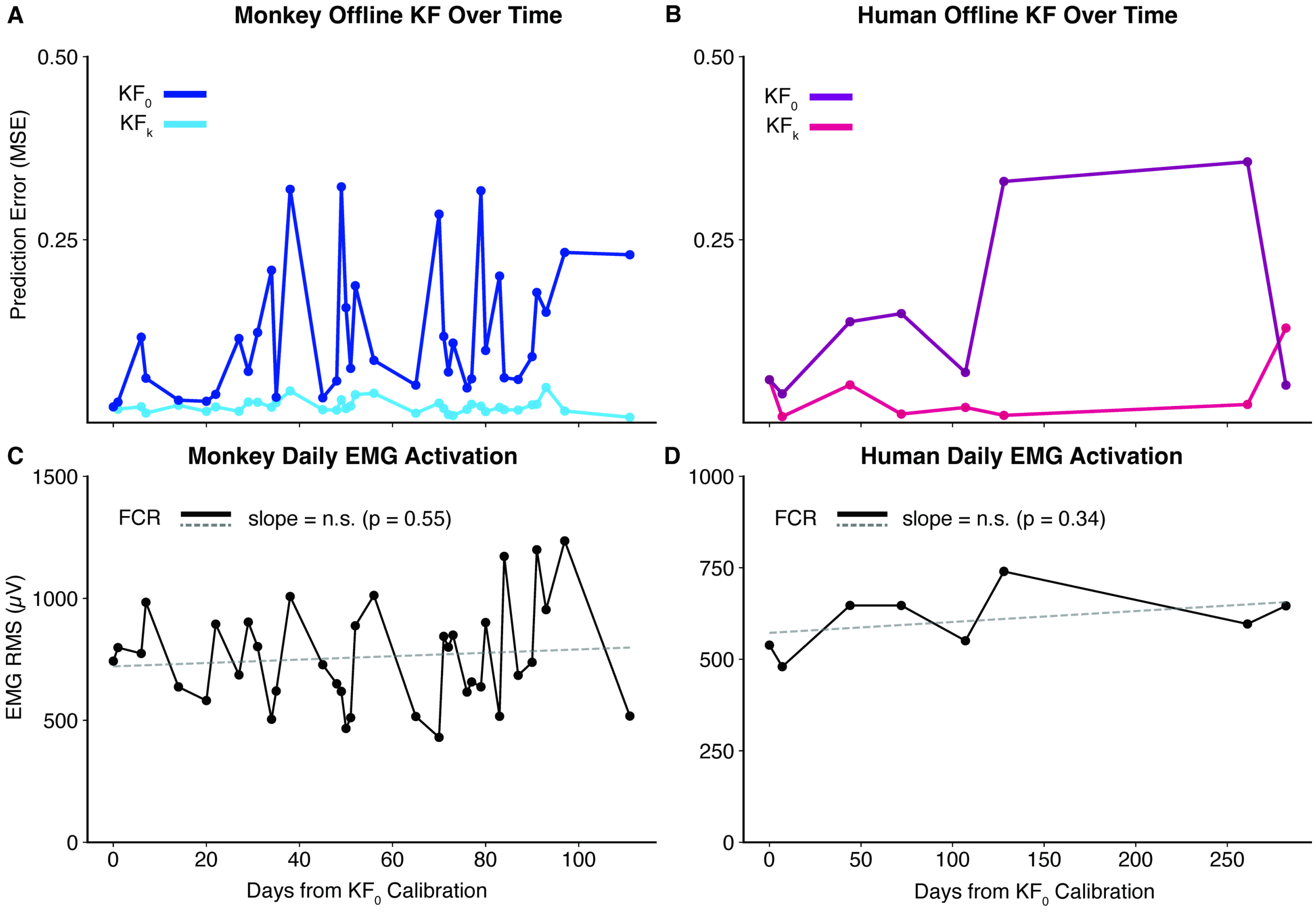

### Figure 4

**Day-to-day variation in offline decoder prediction error and signal amplitude.** (**A-B**) Prediction error (MSE) was measured for both participants' static ($KF_0$) and same-day trained ($KF_k$) decoders when tested on hold-out sets from subsequent online testing days. Significant day-to-day changes in MSE of the $KF_0$ predictions were observed for both participants, while KFk produced predictions with consistently lower MSE. c-d, The RMS of the EMG produced by the participants during all hand-control trials was measured for a major flexor channel (FCR). The linear regression of EMG values over days is indicated by the gray dashed line with the slope and the significance of the slope (p) labeled on the plot.

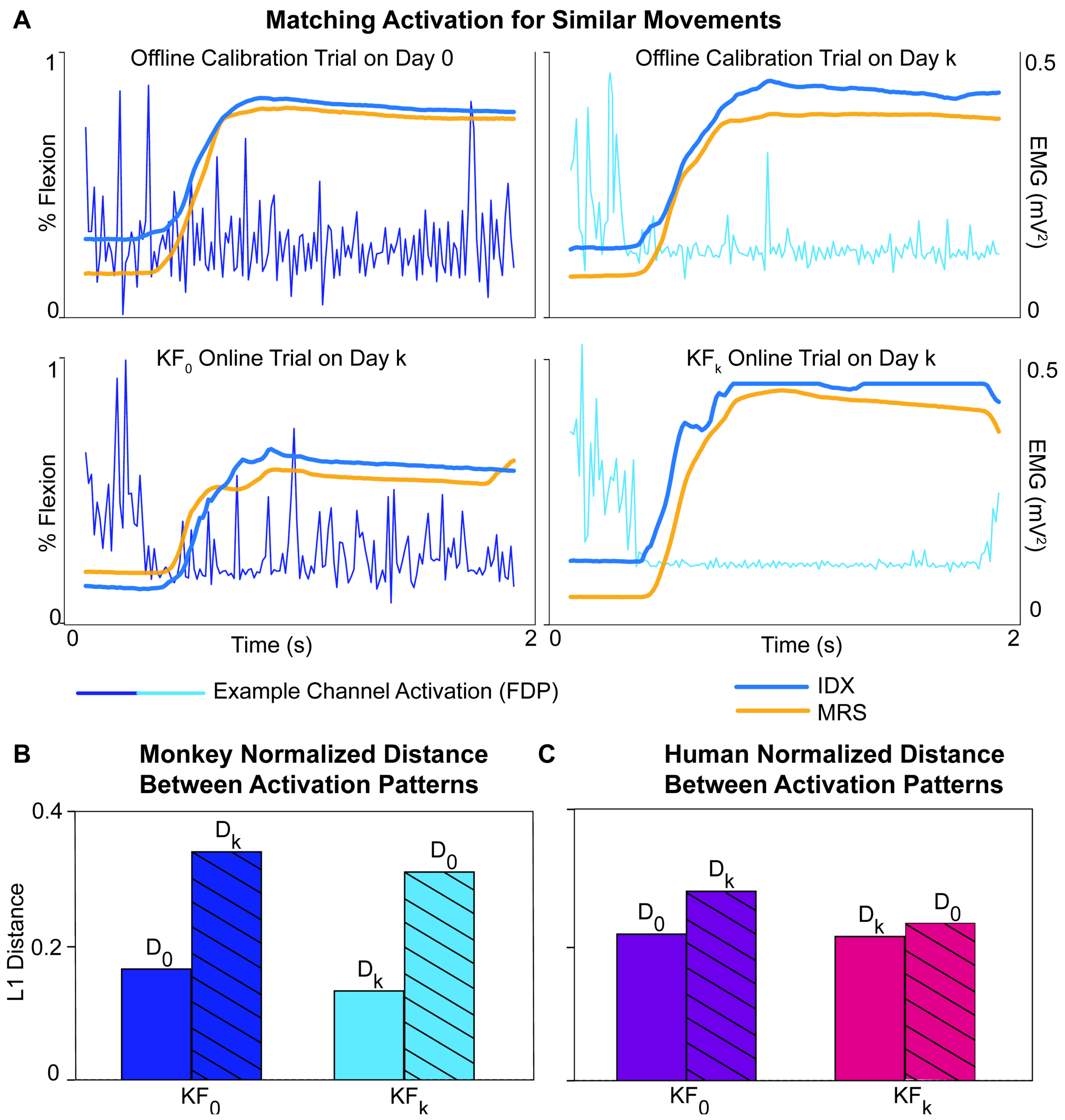

**Figure 5**

**Users adapt their activation to decoder calibration** (**A**) The top row shows example EMG produced on the monkey's FDP during similar dual-flexion (both MRS and IDX) hand-control trials on Day 0 and from a Day k where $KF_0$ had poor offline performance. Hand-control trials on Day 0 were used to train $KF_0$ and hand-control trials on Day k were used to train $KF_k$. The second row shows example EMG on the monkey's FDP on Day k during closed-loop sessions, when he was either using $KF_0$ or $KF_k$ to complete dual-flexion trials. Visually similar activation to Day 0 was observed when $KF_0$ was used online, as was the activation when he used $KF_k$ like activation from Day k calibration trials. (**B-C**) The L1 distance between the pattern of activation from all calibration trials (from Day 0 or Day k) and all closed loop trials (from each Day k where either $KF_0$ or $KF_k$ was used) was calculated across all channels for both participants and normalized by the average activation on each channel during calibration trials. On average, the pattern of activity when the monkey used $KF_0$ was closer to the pattern of activity from Day 0 calibration trials than it was to Day k calibration trials. Similarly, the pattern of activity when the human used $KF_k$ was closer to her pattern of activity from than Day k calibration than from Day 0.

## Supplementary Files

This is a list of supplementary files associated with this preprint. Click to download.

- HumanCrossDayKF.mov
- MonkeyCrossDayKF.mov
- MonkeyCrossDayLSTM.mov
- SupplementalMaterials.docx
